# Cultured Epidermal Melanocyte Transplantation in Vitiligo: A Review Article

**Published:** 2019-03

**Authors:** Shaghayegh ZOKAEI, Dariush D. FARHUD, Mohammad KEYKHAEI, Marjan ZARIF YEGANEH, Hoda RAHIMI, Hamideh MORAVVEJ

**Affiliations:** 1. School of Advanced Medical Sciences, Islamic Azad University, Tehran Medical Branch, Tehran, Iran; 2. School of Public Health, Tehran University of Medical Sciences, Tehran, Iran; 3. Department of Basic Sciences, Iranian Academy of Medical Sciences, Tehran, Iran; 4. School of Medicine, Tehran University of Medical Sciences, Tehran, Iran; 5. Cellular and Molecular Research Center, Research Institute for Endocrine Sciences, Shahid Beheshti University of Medical Sciences, Tehran, Iran; 6. Skin Research Center, Shahid Beheshti University of Medical Sciences, Tehran, Iran

**Keywords:** Vitiligo, Melanocyte, Repigmentation, Melanocyte transplantation, Autologous cultured melanocytes

## Abstract

**Background:**

The color of the skin is highly heritable but can be influenced by the environments and endocrine factors. Many other factors, sometimes destructive, are also involved in the formation of skin color, which sometimes affects pigmentation patterns. Vitiligo is an autoimmune hypopigmentation painless disorder with appearance of white patches and psychological effects on patients. It is a disease in which melanocytes of the skin are destroyed in certain areas; therefore depigmentation appears.

**Methods:**

We studied more than 60 articles. Several therapeutic methods have been used to return the color of skin in vitiligo. These methods include non-invasive treatment and surgical techniques. Among all these therapies, cell transplantation is an advanced procedure in regenerative medicine. Extraction of melanocytes from normal skin and then their cultivation in the laboratory provides a large number of these cells, the transplanting of which to depigmentation areas stimulates the site to irreversibly produce melanin.

**Results:**

The transplantation methods of these cells have been evolved over many years and the methods of producing blister have been changed to the injection of these cells to the target sites.

**Conclusion:**

In this review, autologous cultured melanocyte transplantation has been considered to be the most viable, safe, and effective method in the history of vitiligo treatments.

## Introduction

### Skin Structure and Function

**Fig. 1:**
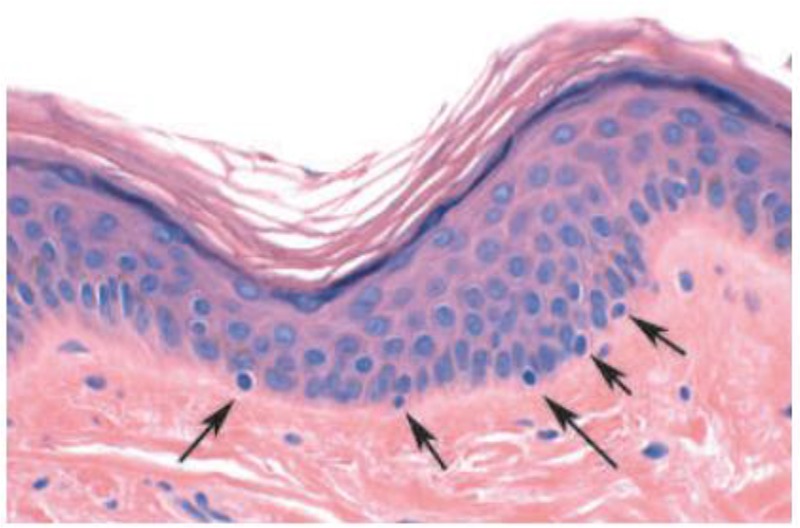
Haematoxylin and eosin stain of normal human skin. Dermis, muscle and nerve fibers appear pink. Melanocytes, with small nuclei, are located in the basal layer of the epidermis, at the junction with the dermis

The skin is the largest organ of the body, protecting it from the external hazards as a static barrier and functioning as a sensory organ. It accounts for about 15% of the total adult body weight with the thickness varying from 1 to 4 mm (1–3), covering an area of approximately 1.4∼2.0 m^2^ (4). Loss of skin integrity may cause substantial physiologic imbalance and significant disability or even death. The skin consists of three layers, from top to bottom: the epidermis, dermis, and hypodermis. Epidermis with the thickness of 100–150 μm, is the most superficial and biologically active layer of the skin. It is known to be composed of about 95% keratinocytes (of which the lowermost are anchored to the basement membrane via hemidesmosomes), melanocytes, Langerhans cells, and Merkel cells (mechanoreceptors). Dermis is separated from the epidermis by the dermal-epidermal junction. It is highly vascular and consists of the pilosebaceous units, sweat glands, dermal adipose cells, mast cells, fibroblasts, infiltrating leucocytes, and connective tissue elements including collagen, elastin, glycosaminoglycan, collectively termed the extracellular matrix (ECM). With the thickness of 2–4 mm, dermis provides most of the mechanical strength to the skin. Hypodermis is composed of subcutaneous fat (2, 3, 5) (Fig. 1).

Keratinocytes cells are the upper layer of the epidermis contain larger nuclei and stain blue (6).

### Skin Color

Skin color, ranging from white to black, is one of the most important factors in the beauty, determined by the combination and distribution of different chromophores, one of which is melanin. Melanin, produced by melanocytes, is the major dark pigment found in skin, hair, and eyes that provides protection against aging and carcinogenic effect of ultraviolet radiation. The amount and distribution of melanin in the pigmentation process are influenced by factors such as genetics, environment and endocrine factors (7, 8).

### Melanocytes

Skin melanocytes (MCs), the density of which reaches 500–2,000 cells per m*m*^2^ of cutaneous surface, are localized in the basal layer of the epidermis and hair follicles (1). Each melanocyte is surrounded by approximately 4–10 basal keratinocytes (9). Melanocyte’s dendrites expand between keratinocytes and KCs-derived growth factors stimulate proliferation and differentiation of MCs (10). One of the functions of melanocytes is pigmentation through the production of melanin pigments, produced in melanosomes and can be transferred to the keratinocyte and stored there. Although melanin is mixed with other pigments such as carotenoids and hemoglobin derivatives to compose the skin color, it is the principal pigment of the skin that can be found in two different colors: yellow/red (pheomelanin) and brown/black (eumelanin). People with more pigmented skin have less risk of developing skin cancer or sunburn because eumelanins are more photoprotective than pheomelanins (10–12).

### Melanogenesis

**Fig. 2:**
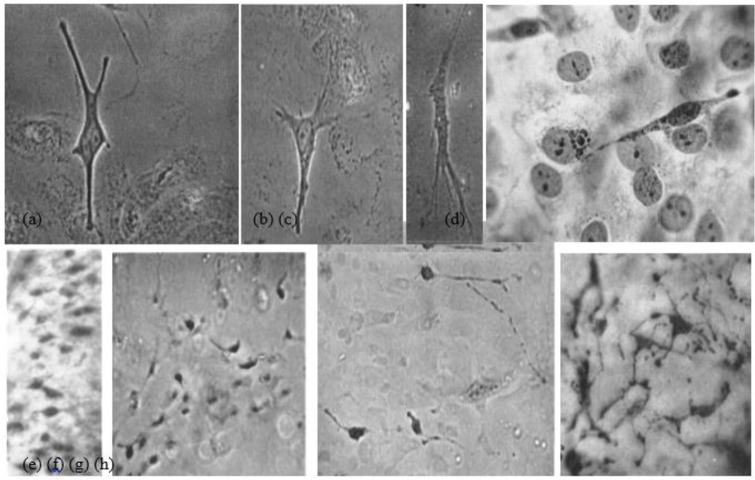
(a, b, c) Cultures of pigmented nevi: Melanocytes with the granular cytoplasm, and dendritic processes with secondary branching. (d) A bipolar melanocytes with one process. (e, f, g, h). Dopa reaction. Melanocytes in cultures of white and pigmented foreskins with different sizes and shapes. (e & f). Dopa-positive cells in white foreskin cultures. (e) Melanocytes in the split portion of original explant (f) and in the outgrowing sheet of same. (g & h). Darkly pigmented foreskin. (g). The pigment cells in the outgrowing sheet of dark skin explant with the same size and shape (Source: Reference 12)

**Fig. 3:**
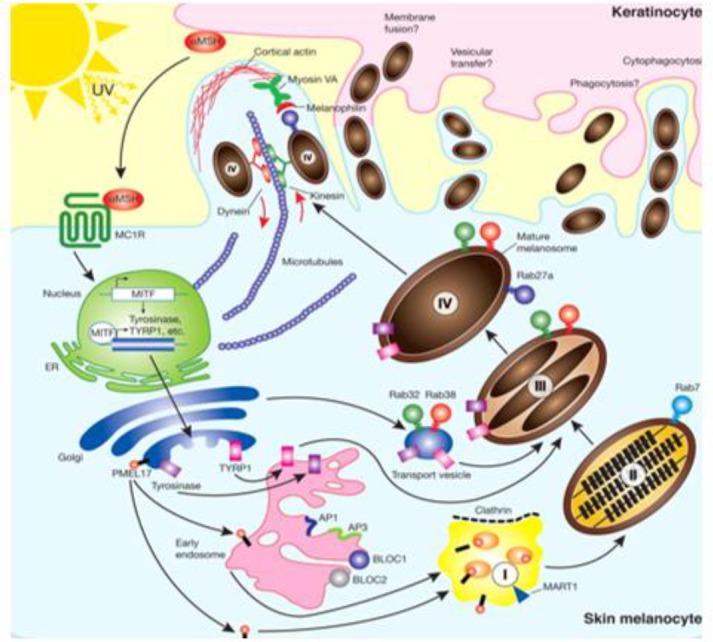
Melanogenesis. The pathway for melanin synthesis (Source: Reference 15)

Melanogenesis is the process of making melanin, which requires three enzymes for its proper activity: tyroninase, TRP1 (tyrosinase-related protein 1) and TRP2 (tyrosinase-related protein 2). Tyrosinase catalyzes the first two reactions of the biosynthesis of melanin that are necessary for producing eumelanin and pheomelanin, while TRP1 and TRP2 are only involved in the pathway of eumelanin synthesis. Tyrosinase uses tyrosine, DOPA and 5, 6 dihydroxyindole (DHI) as substrates to produce respectively DOPA, DOPA-quinone and DHI-melanin. Tyrosinase activity is regulated by some factors such as the pH (optimal at 6.8 in melanosomes) and melanocyte-stimulating hormone (α-MSH) (10). When α-MSH binds with melanocortin receptor-1 (MC1R), eumelanin pigments will be produced, whereas when α-MSH does not recognize MC1R, pheomelanin pigments are generated (6, 10). Following this production of melanin, the melanosomes which have pigments are transported towards the end of the melanocyte dendrites by actin and tubulin filaments. Then, melanosomes would be transferred to keratinocyte (10, 13–15) (Figs. 2, 3).

### Keratinocytes

Keratinocytes, an impermeable barrier to pathogens, play an important role in cell signaling within the extracellular matrix (16). The morphology and differentiation degree of KCs varies with the epidermal layer where they are observed. Human keratinocyte growth factor (KGF), an epithelial cell-specific mitogen, is secreted by normal stromal, which causes melanocytes grew well in medium when they cocultured with keratinocytes (17, 18). KGF acts in a paracrine manner to promote epithelial cell growth and wound healing (19).

### Pigmentation Diseases

Any disorders in the synthesis of melanin or melanocytes may lead to various skin pigmentation pathologies. Some disorders are characterized by the presence of dark patches on the skin (hyper-pigmentation), while others are recognized by loss of skin pigmentation (hypo/depigmentation) (10). These hypo/depigmentation disorders, lead to white macules/patches, may be caused by the destruction of melanocytes, inhibition of development of melanocytes, or prevention of melanin production. Vitiligo is characterized by the first mechanism, piebaldism by the second, while oculocutaneous albinism and tinea versicolor are characterized by the third mechanism (10–14,16–19).

### Vitiligo

**Fig. 4:**
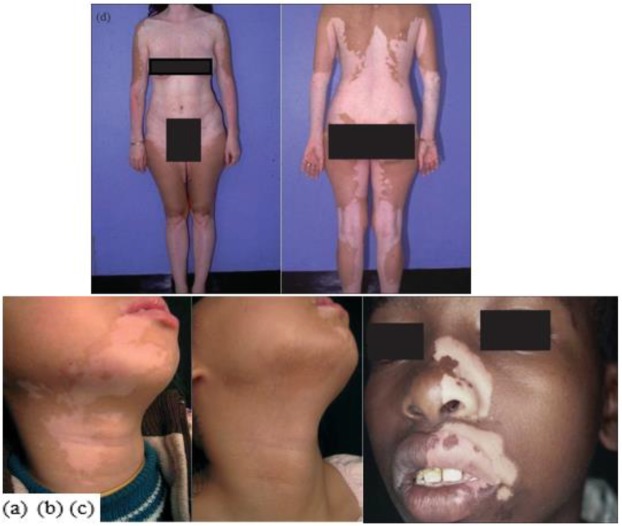
(a) before and (b) after the transplantation of autologous cultured melanocytes on the neck and chin (22); (c) Segmental vitiligo on one side of the face. (d) Symmetrical distribution (or non-segmental) vitiligo (Source: Reference 20)

Vitiligo is an autoimmune hypopigmentation disorder which is complex and characterized by patchy loss of skin pigmentation and destruction of functional melanocytes in the epidermis which can affect any part of the body that has pigmented cells (20–22). Several mechanisms have been proposed for pathogenesis of vitiligo including autoimmunity, neural theory, and oxidative stress (23). Two types of vitiligo have been observed: Segmental and non-segmental (or Symmetrical). Segmental vitiligo, which occurs most commonly at an early age, manifests in one segment of the body (e.g., a hand, a leg, or the face), however, non-segmental vitiligo, which is more common, affects both sides of the body in a confined area (20) (Fig. 4).

### Treatments

Various treatment modalities have been developed for repigmentation of vitiliginous skin (10, 21, 22, 24). These methods include non-invasive treatment and surgical techniques. The noninvasive treatment used for vitiligo includes psoralen plus ultraviolet A (PUVA), narrowband ultraviolet B (NB-UVB), excimer lasers, topical steroids, topical immunomodulators, and calcipotriol. Lack of response to these non-invasive treatments is common in different sites of the body (e.g., hands and feet); therefore, over the years, many surgical techniques have become available for achieving repigmentation in vitiligo divided into tissue and cellular grafting. In these techniques autologous melanocytes obtained from a small and normal donor skin biopsy are transplanted to the depigmented area; furthermore, the injection of epidermal cells into skin blisters can be used for small areas and cultured epidermal autografts have been used for larger areas. Cellular transplantation includes cultured pure melanocytes suspension and non-cultured epidermal cellular suspensions. These techniques have both advantage and disadvantage. The advantage is that these methods, unlike the tissue graft, allow to treat damaged skin manifold larger than the donor sites. However, they are almost costly and time-consuming because of the several weeks required for culturing time, and also require a specialist, fully trained staff, and well-equipped tissue laboratories; however, it has been reported that transplantation of autologous cultured melanocytes successfully repigment vitiliginous skin (22,24–30).

### Cell Culture Process

**Fig. 5:**
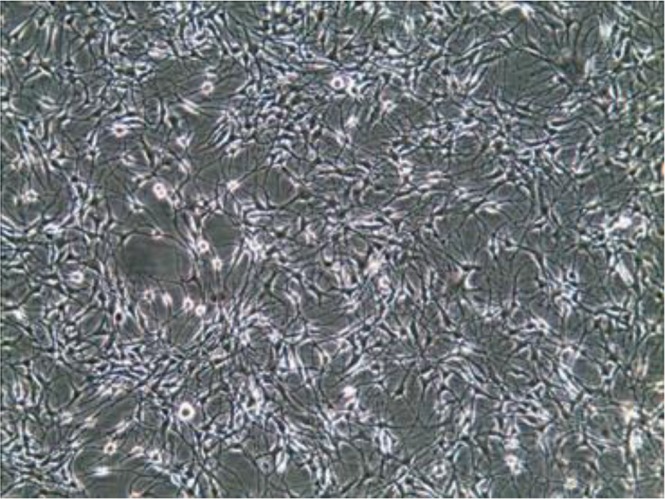
Cultured melanocytes in vitro (Source: Reference 22)

Morphology of melanocyte cells in culture Autologous cultured melanocyte transplantation is viable, safe, and effective (31). For the first time, in 1956, it was worked on human melanocytes of benign pigmented nevi and foreskin of white and black infants using tissue culture method and indicated the presence of two distinct types of cells in normal human epidermis: epithelial cells and melanocytes, which differ morphologically, functionally and biochemically. Moreover, two types of melanocytes observed, the small type as ordinarily seen in normal epidermal outgrowth and a large variety and the latter was at least 2 to 4 times larger than the small type, reacted strongly to DOPA reagent and became filled with black granules. There was no apparent difference in the number of melanocytes found in cultures of white or colored skins, but the number of melanin granules, sizes, and shapes of melanocytes in cultures of white and pigmented foreskins, were different and also, there appeared to be a direct relationship between pigment-producing capacity and cellular size and complexity (12) (Fig. 5).

### Skin Specimens

**Fig. 6:**
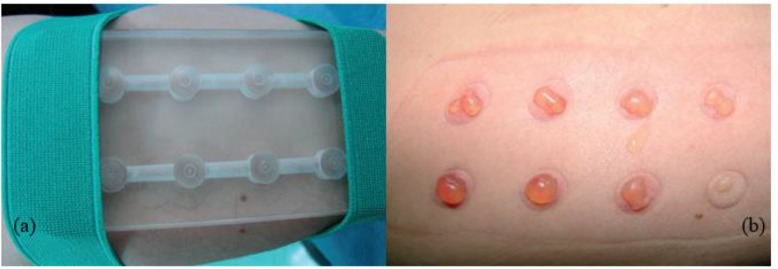
(a) A suction blisters-forming dish. (b) Blisters on the forearm (Source: Reference 35)

First of all, skin samples should be obtained from the non-cosmetic and pigmented areas like the buttocks, thighs, forearm or waist. These specimens can be both roofs of blisters (32) or a superficial shave biopsy gained with a silver’s skin grafting knife or a skin razor blade (33). The blister can be created either by a suction device, which was first a foot-operated suction blister machine used in India (34), or liquid nitrogen (32). To make blister with the device, the skin specimens can be warmed with a thermophore and also syringes, plastic cups, and funnels connected to a vacuum suction machine are used (32, 35) (Fig. 6).

### Separation

Trypsin, Collagenase, and Dispase are enzymes to separate epidermal tissue from the dermis. There had been no reports of appropriate separation between adult epidermis and dermis and eventually successful pure cultivation of epidermis until 1960, when Cruickshank and partners indicated that in progressive vitiligo an active depigmenting mechanism often prevents repigmentation and described a method for culturing adult epidermal cells based on preparing a cell suspension by using trypsinization and subsequent cultivation in a simple chamber with good microscopic properties. Both epithelial and dendritic cells were obtained and multiplied, reducing the risk of fibroblast presence (36). Lots of efforts were also made to separate epidermal cells enzymatically, for instance, using collagenase to separate epidermis from dermis. For this purpose, in a study, small split skin pieces incubated in collagenase were used, and after incubation, the epidermis was peeled off in sheets and finally dissociated by trypsin-EDTA. During the first days of the cultivation, the cells did not have homogeneous morphology and the epidermal cells appeared mainly as polygonal cells of various sizes and a few little dendritic cells (37).

The problems in cultivation of isolated melanocytes were expressed: separation of epidermal cells, purification of the melanocyte culture, and promotion of sustained growth of the melanocytes. Trypsin flotation has an advantage over collagenase treatment because it produces more viable and purer melanocytes. Three methods of separation were presented: In the first method, human skin obtained from the thigh or breast of patients and incubated these skin samples in collagenase. Then the epidermis separated from the dermis and incubated epidermis in dithioerythritol. All samples were transferred to a trypsin solution and shook. In the second method, skin samples floated on trypsin. After that, epidermis, separated from the dermis, was dissociated into a single cell suspension by pipetting. In the third method, the growth of keratinocytes prevent by seeding non-separated single-cell suspensions in the culture medium without Mg^2+^ and Ca^2+^ (38). Moreover, another study referred to the disperse enzyme, proven to work intensely and effectively (38, 39).

### Isolation and proliferation

To isolate melanocytes under culture conditions, trypsin was used in high levels of mycostatin (containing nystatin, which is less toxic for eukaryotic cells) to increase the intracellular calcium concentration, then to inhibit keratinocyte adhesion and eventually to produce pure melanocyte with an estimated purity of 95%. Within 48 h to one week of cell culture, melanocytes first appeared as dark dendritic cells and then all rounded cells were depleted. The addition of high concentrations of Mycostatin to growth medium produced relatively pure, normal and viable melanocyte populations (40). Some years later, especially proliferation, culture, and passages were studied with different kinds of media and growth factor. On this way, combination of factors including cholera toxin (which inhibits the growth of fibroblasts) and phorbol 12-myristate 13-acetate were tested in the culture medium, which is toxic to human keratinocytes but not to melanocytes, pH 7.2, and fetal calf serum at 5% rather than at 10%. In this way, melanocytes can proliferate extensively and passage serially in vitro (41). However, one year later, series was used of phorbol esters, teleocidin, and aplysiatoxin, which has tumor-promoting activity and also are potent enhancers of the growth of human melanocytes (42). In these methods, qualified epidermal melanocyte cells from skin had been achieved with high purity.

### Medium and factors

Cells from the normally pigmented skin of the shoulder using MCDB-153 medium was studied and grew them on collagen-coated substrate. Melanocytes grew well in the MCDB-153 medium when they cocultured with keratinocytes (because of growth factors produced by keratinocytes), and dendrites of the melanocytes were attached to neighboring keratinocytes, and also fibroblasts do not proliferate in the MCDB-153 medium (43). In another method of cultured epithelial grafts, in previous methods, cytotoxic agents and TPA, a potent tumor promoter, were used and also cells from these patients cannot grow well in the presence of TPA. However, in this study, pigmented epidermal cells from vitiligo patients were cultured on a collagen-coated membrane in MCDB-153. Then, the cell/collagen-coated membrane was used to cover a superficially dermabraded vitiliginous area (44). Moreover, in a study on nine patients with stable vitiligo, the H-MEM was used, which is another form of Eagle’s Essential Medium with Hanks’ Salt and has a higher concentration of necessary amino acids, sodium bonds, and nucleic acid precursors, without using any growth enhancers or hormones. A high percentage of success was observed in the outcome of the treatment (45). To increase the number of melanocytes in culture, Endothelin 3 was used. The presence of Endothelin 3, which is a potent mitogen and acts in a synergistic manner with factor(s) found in CEE plus FCS-containing medium, promotes NC cell (neural crest cell which differentiates into a variety of cell types including melanocytes) and ultimately increases melanocytes. From the first day of culture to the last, they analyzed the effects of EDN3 with different concentration levels in medium, and demonstrated a change in cell morphology, an increase in cell number and in the number of differentiated melanocytes, in high concentration levels of EDN3 compared to the control group (46). However, instead of all chemical mitogens, Gábor Szabad et al used an autologous human serum (AHS). In most of melanocyte cultured methods, chemical mitogens or growth factor supplement were used, for example, the use of EGF, BPE, and FBS in medium by Donatien (47), or bFGF, ET-1 and ∝-MSH- utilized by Swope VB (48), to increase melanocyte growth. In their method, they used an autologous human serum (AHS), as basal medium and superior to FBS, Keratinocyte Basal and epidermal growth factor (EGF), which can increase the melanocyte proliferation, without the presence of chemical mitogens. Autologous human serum alone could provide sufficient growth support for cells grown in the basal medium and normal human adult melanocytes expressed both EGF receptor (EGFR) mRNA and protein. Nevertheless, after several passages without chemical mitogens, melanocytes lost pigmentation and TRP-1 expression (49). In addition, Adipose-derived Stem Cells (ADSCs) can be used as a substitute for Keratinocytes in co-culturing. In another method, to examine the effect of ADSCs or keratinocytes, they seeded ADSCs or keratinocytes into a plate. After 24 h, they removed supernatant and added human melanocytes suspended in the medium into that plate. By using immunohistochemistry and Boyden chamber cell migration assay, they compared proliferation, differentiation and, migration of melanocytes in the presence or absence of those feeder cells. ADSCs, like Keratinocytes, have an effect on increasing melanocyte proliferation and migration because of producing growth factors, more at 2–3 wk while reducing differentiation and also being less powerful than keratinocytes. Thus, this method turned out to be effective in treating vitiligo with melanocyte cell culture (50).

### Injection techniques and Transplantation

**Fig. 7:**
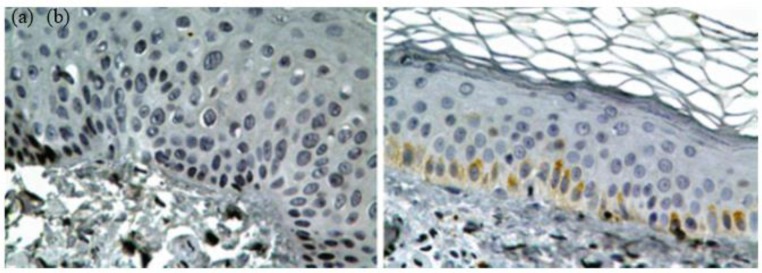
skin bopsy. Epidermal cells (a) before and (b) after transplantation (Source: Reference 56)

To transplant the melanocyte cell, first the recipient sites should be prepared. Different methods have been reported such as dermabrasion, blister (suction or liquid nitrogen), and injection. In dermabrasion method, first, the vitiliginous area should be surgically cleaned and locally anesthetized. Then, the recipient sites are dermabraded down to the papillary dermis, using dermabrader and finally cell suspension is applied uniformly on the denuded area (51). To obtain blisters, different methods were used, for instance using an electric vacuum suction machine to make blisters (52) or using nitrogen gas, which is less painful, to freeze multiple spots, then within 24 to 48 h, blister occurred so denuded area was ready for grafting. The gauze carrying the epithelial graft placed on the denuded spots (45, 52). Moreover, at the stage of transplantation melanocytes can be covered with a collagen dressing and gauze (28). Moreover, to prepare recipient sites, a carbon-dioxide laser was used to remove the epidermis of the recipient site, and then the melanocyte suspension was applied to the area (53). In a remarkable method, amniotic membrane (AM) was used as a scaffold for melanocyte transplantation in 4 patients with both stable generalized and focal vitiligo. To use Amniotic Membrane, they first obtained Placentas during cesarean delivery. After that, the amnion was separated from chorion by blunt dissection, and then in sequence, the membrane was flattened, cut up, thawed and washed. All melanocyte cells were digested with trypsin and EDTA and then replated into the basement membrane side of AM and were cultured for 3–4 more days. Then to prepare the recipient sites they used the Silk Touch Flash-scanner attached to a Sharplan 1030 CO2 laser and the denuded skin was treated with the AMs containing cultured melanocytes. They represented that this culture method on AM as a scaffold is a unique, simple and successful treatment (54). Another technique is injection, which a dermatologist injects the cell suspension, using a needle. Unlike other transplantation methods with severe complications and pain, mild erythema and swelling in the recipient areas was observed in this method (55) (Fig. 7).

### Cryostorage

To multiply and reuse the excess of melanocytes cells, Olsson MJ et al were working on melanocyte cell culture storage techniques. In repigmentation with cultured melanocytes techniques, on a small specimen of pigmented buttock skin, they used cryostorage for 6–12 months for the next treatment of those patients and after one week of reculture, reimplanted into vitiliginous areas (57).

### Age effect

A comparative study was conducted on vitiligo treatment using autologous cultured pure melanocytes transplantation among children, adolescents, and adults with localized vitiligo. They isolated and transplanted melanocyte suspension. The result of the cell culture transplantation technique in children and adolescents was not only comparable to the adult, but also better, and no statistical difference was seen in the result of repigmentation. Therefore, the technique was suitable and effective for children and adolescents, too (58).

### Responders and Nonresponders

To find out why there is a lack of proper result in repigmentation of some patients, a different study was done. Unlike patients with piebaldism who suffers from a lack of pigmentation from the beginning of their life, vitiligo patients have immunological destruction that can change the outcome (59). A comparative study was undertaken by A. Rao et al about the clinical stability of generalized vitiligo among 3 groups depending on the elapsed days of the increasing size or appearance of the last lesion, and its relation with Catalase levels and immunohistochemistry of CD4, CD8, CD45RO, CD45RA and FoxP3 levels between the responders and nonresponders. Between-group 1, 2, and 3, responders to melanocyte transplantation had a higher period of stability, lower CD8 count and complete absence of CD45RO in comparison with the nonresponders and they also stated that no difference was observed in CD4, CD45RA, FoxP3, and blood catalase levels between the responders and nonresponders. Therefore, to determine vitiligo stability and obtain the best result in melanocyte transplantation, the percentage of CD8 and CD45RO cells is helpful (60). So patients with active vitiligo have a poor response to transplantation, because of melanocyte-destroying factors that are present and active, while patients with stable localized vitiligo and stable generalized vitiligo have a great outcome (54). Vitiligo patients with hypothyroidism or widespread vitiligo respond less well to the transplantation method and should not be treated with transplantation (61). On another hand, these are related to the amount of antibody which is effective in responding to therapeutic approaches, because the high level of antibody in active or progressive vitiligo prevents the appropriate response. Furthermore, the immunofluorescence method was used to detect antibody located in the cytoplasm of melanocytes, so the stable stage and the developmental stage of patients could be discovered with this testing of melanocyte antibody (62).

### Culture or Non-culture

To evaluate and compare the effects of culture and non-culture (autologous melanocyte rich cell suspension) techniques, different studies were done in which they referred to a vast coverage of vitiliginous areas in culture technique (33). In one of these comparative studies, they pointed out in NCMT method, they incubated the mixture of skin sample and trypsin-EDTA solution and transferred them to the medium. However, in CMT method, they cultured that cell suspension in tissue culture flasks and after 21 d the number of melanocytes was counted. According to this study, there was no statistical difference between these two groups, and it was found that both therapeutic projects were beneficial in more than 50% of the cases, but inferior result in CMT method was seen because of the delay in transplantation. However, they used 1 cm2 of normal skin to cover about 100 cm2 of vitiliginous skin, compared to NCMT in which 1 cm2 of normal skin is used for about 10 cm2 of vitiliginous skin (51). Moreover, referring to another study of re-pigmentation in cultured and non-cultured melanocytes transplantation, the use of a small area of donor skin to cover an enlarging area is the most important indicator. In their study, similar lesions of the same cases were transplanted with either NCES or CMT and three diagnostic methods were used: visual, 3-D, and 2-D. By following 27 out of 30 people, more satisfaction of patients and more than 50% of better repigmentation were observed in sites treated with CMT compared to NCES (63).

## Conclusion

Cellular transplantation has been a unique surgical technique in the last few decades to treat stable vitiligo in patients not respond to different therapies such as pharmacologic therapy, immunotherapy, phototherapy, photochemotherapy, and mini grafting. In many studies, more than 50% success has been observed, except for poor results in fingers, knees, and elbow areas. Sustainability of this disease is an important factor in using this method because the presence of stimulant factors leads to a lack of proper response to this therapeutic approach.

In this method, melanocytes are isolated from normal human skin and cultured in the medium then transplanted to recipient vitiliginous area, so we can cover large vitiliginous areas by using only a smaller donor skin, unlike the non-culture method that covers more limited parts. Moreover, today due to the newer methods of sampling and transplantation, the complications of this therapeutic approach are less, for example, using lasers or syringe injection. There is no significant and statistical difference in this method of treatment between children and adults, so we can use this method for both groups. However, it is still possible to consider cultured melanocyte transplantation as the most viable method for the treatment of vitiligo.

## Ethical considerations

Ethical issues (Including plagiarism, informed consent, misconduct, data fabrication and/or falsification, double publication and/or submission, redundancy, etc.) have been completely observed by the authors.
